# Quantifying the Validity of Routine Neonatal Healthcare Data in the Greater Accra Region, Ghana

**DOI:** 10.1371/journal.pone.0104053

**Published:** 2014-08-21

**Authors:** Gbenga A. Kayode, Mary Amoakoh-Coleman, Charles Brown-Davies, Diederick E. Grobbee, Irene Akua Agyepong, Evelyn Ansah, Kerstin Klipstein-Grobusch

**Affiliations:** 1 Julius Global Health, Julius Center for Health Sciences and Primary Care, University Medical Centre Utrecht, Utrecht, The Netherlands; 2 Ghana Health Service, Greater Accra Region, Accra, Ghana; 3 School of Public Health, University of Ghana, Legon, Accra, Ghana; 4 Division of Epidemiology and Biostatistics, School of Public Health, Faculty of Health Sciences, University of the Witwatersrand, Johannesburg, South Africa; UCL, United Kingdom

## Abstract

**Objectives:**

The District Health Information Management System–2 (DHIMS–2) is the database for storing health service data in Ghana, and similar to other low and middle income countries, paper-based data collection is being used by the Ghana Health Service. As the DHIMS-2 database has not been validated before this study aimed to evaluate its validity.

**Methods:**

Seven out of ten districts in the Greater Accra Region were randomly sampled; the district hospital and a polyclinic in each district were recruited for validation. Seven pre-specified neonatal health indicators were considered for validation: antenatal registrants, deliveries, total births, live birth, stillbirth, low birthweight, and neonatal death. Data were extracted on these health indicators from the primary data (hospital paper-registers) recorded from January to March 2012. We examined all the data captured during this period as these data have been uploaded to the DHIMS-2 database. The differences between the values of the health indicators obtained from the primary data and that of the facility and DHIMS–2 database were used to assess the accuracy of the database while its completeness was estimated by the percentage of missing data in the primary data.

**Results:**

About 41,000 data were assessed and in almost all the districts, the error rates of the DHIMS-2 data were less than 2.1% while the percentages of missing data were below 2%. At the regional level, almost all the health indicators had an error rate below 1% while the overall error rate of the DHIMS-2 database was 0.68% (95% C I = 0.61–0.75) and the percentage of missing data was 3.1% (95% C I = 2.96–3.24).

**Conclusion:**

This study demonstrated that the percentage of missing data in the DHIMS-2 database was negligible while its accuracy was close to the acceptable range for high quality data.

## Background

Data quality assurance is of high priority in any clinical research because the quality of the data is a major determinant of the validity of the conclusions drawn. High quality data can be ensured by adherence to Good Clinical Data Management Practice (GCDMP) [1] although there is no consensus on what should be regarded as guidelines for GCDMP for all the fields of healthcare research [2]. Generally, it is believed that the information on regulations and guidelines for GCDMP can be obtained from the International Conference on Harmonisation (ICH), Food Drug Agency (FDA) and Society for Good Clinical Data Management [3]–[5] to inform formulation of Standard Operating Procedures (SOPs) for data collection.

Data can be captured by Electronic Data Capturing (EDC) and Paper-based Data Collection (PDC) method. Both methods are prone to errors thus, careful assessment of data quality prior to analysis is essential. Errors can be detected in clinical data by double data entry, logic check (range check, detection of outliers, relational conflicts and more) and visual verification [6]. All these methods have their own limitations. The quality of a dataset can be quantified by estimating its accuracy (error rate) and completeness (% of the missing data) [6]. Validation of PDC is usually done by comparing the Case Report Form (CRF) to the database even though this is not in accordance with Good Clinical Practice recommendations [7]. Ideally, in data validation, the database should be compared to the data source i.e. patient's folder or register (primary data source) [7] to avoid the underestimation of the error rate as previously reported by Nahm and colleagues [8]. In the CRF - database validation, some data collection processes that precede CRF will not be examined. Routine clinical data collection in low and middle income countries (LMICs) are mostly paper-based before uploading them to the database.

Similar to other LMICs, routine clinical data in Ghana rely on PDC and prior to May 2012, the District Health Information Management System (DHIMS) was used to manage routine data collected by the health facilities. Health facilities collated and forwarded their data to the districts. The district offices further collated and forwarded the data to regional and subsequently national level before data were uploaded to the national database (data acquisition process). Recently, a web-based database called DHIMS-2 was launched with the aim of improving the quality of the DHIMS data by shortening data acquisition processes. This new method still relies on PDC but the number of data acquisition processes have considerably reduced suggesting possible improvement in the quality of the DHIMS-2 database compared to the previous DHIMS database [9], [10].

Further, the new data management system allows health facilities to collate and upload their data directly to the DHIMS-2 database with instant access at the district, regional and national level. Small health facilities that lack internet facilities and manpower to upload their data to the database continue to forward data to the district office for uploading. This is a great achievement when compared to the former system of handling data (DHIMS). Despite the introduction of this new improved data management system, the quality of the DHIMS-2 data has not been assessed in detail. Thus, this study aimed to quantify the quality of routine neonatal data in the DHIMS-2 database by evaluating its accuracy (error rate) and completeness (% of missing data) for subsequent use in clinical research, evidence-based health policy formulation, and monitoring progress towards attaining Millennium Development Goal 4 (MDG 4 aims to reduce under-five mortality by two-thirds between 1990 and 2015).

## Methods

### Setting

This study was conducted in the Greater Accra Region (GAR), one of the ten administrative regions of Ghana. The region is located in the southern part of Ghana with a population of about 4 million [11] and a neonatal mortality rate of 21 per 1,000 live births [12]. The GAR has ten administrative districts: Accra Metropolis, Ga South Municipality, Dangme East District, Dangme West District, Tema Metropolis, Ledjokuku-Krowor Municipality, Ashaiman Municipality, Adenta Municipality, Ga East Municipality and Ga West Municipality. Communities in this region are mostly urban and the region is served by both public and private health facilities. The DHIMS-2 database covers all the public and few private health facilities.

### Design of data collection

**Figure 1:**
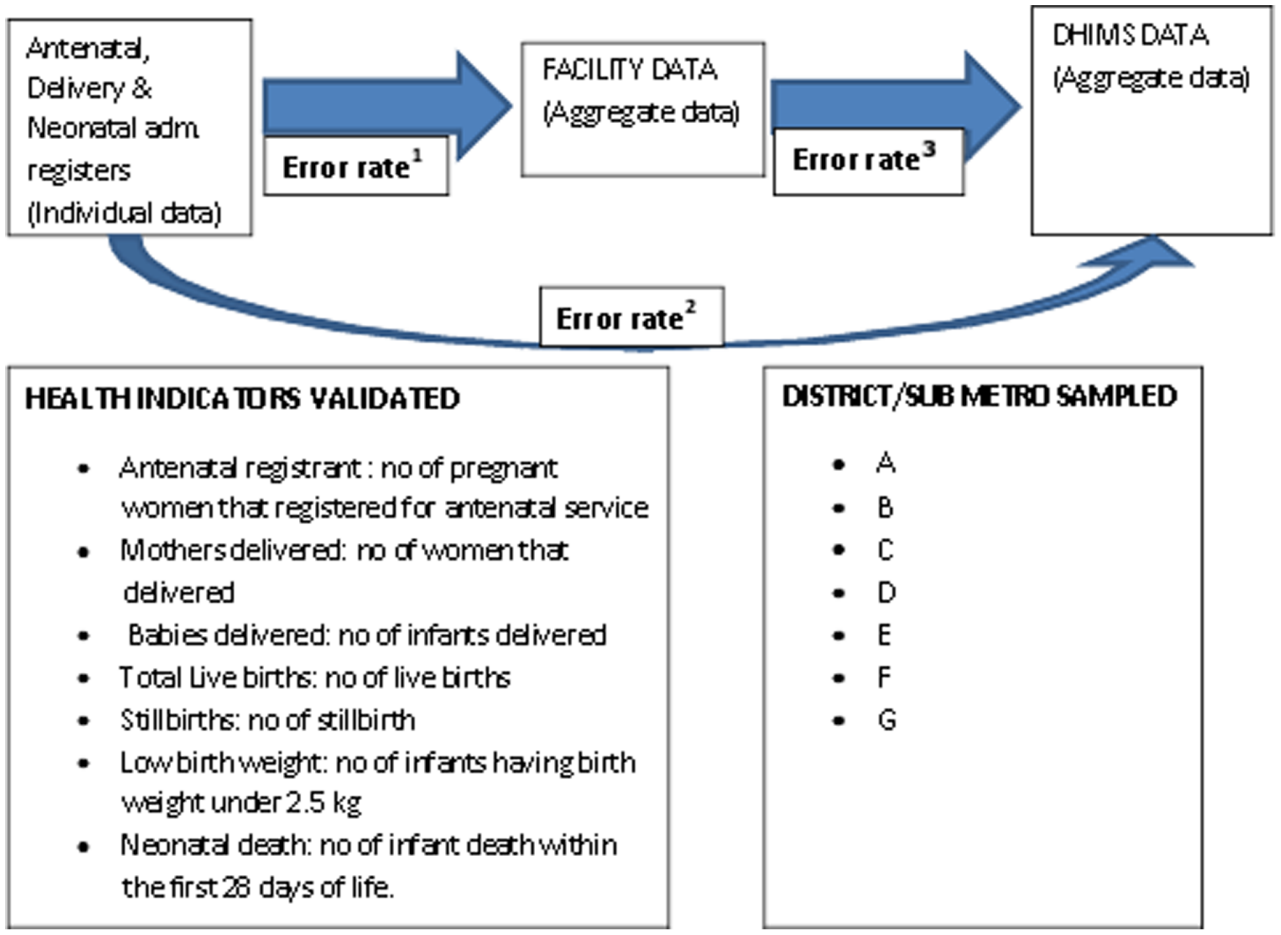
Data flow from the primary data sources to DHIMS-2 database.

Collection of data to validate the DHIMS-2 database was carried out in the GAR. Given the financial limitations, data collection could not be extended beyond the GAR. Seven out of the ten districts in the GAR were randomly sampled for inclusion in the study; we anonymized the sampled districts as district A, B, C, D, E, F and G. The district hospital (secondary level of care in low resource setting) and a polyclinic (primary level of care in low resource setting) in each of the sampled districts were recruited for the study and where one of these health facilities was not available, a health centre (primary level of care in low resource setting but smaller than a polyclinic) in that district was considered. Seven neonatal health indicators were pre-specified for validation: antenatal registrants, deliveries, live birth, stillbirth, low birth weight and neonatal death. Data captured on these health indicators during the first quarter of 2012, were retrieved from thirteen health facilities in the sampled districts with the support of trained research assistants who collected information in a standardized manner. We examined all the data captured on the pre-specified health indicators during the first quarter of 2012 because all the districts have uploaded the data captured during this period to the DHIMS-2 database. Data were retrieved from the primary data sources (antenatal, delivery and neonatal register), facility data and DHIMS-2 data. Antenatal, delivery and neonatal registers are paper register where clinical and non-clinical profiles of the patients are recorded when they present at antenatal, delivery or neonatal intensive care unit. Data were extracted from the primary data sources on the pre-specified health indicators and the differences between the estimated values of the health indicators obtained from the primary data and that of the facility and DHIMS–2 data were used to estimate the accuracy of the DHIMS-2 database. Completeness of the DHIMS-2 database was estimated by calculating the percentage of missing data in the primary data. Primary data (individual patient data) were obtained from the antenatal, delivery and neonatal register while the facility and DHIMS-2 data (aggregate data) were provided by the health facilities and the Biostatistics Department of the Greater Accra Regional Health Directorate respectively. In addition, semi-structured questionnaires were used to gather information on the data acquisition processes as shown in Figure 1.

### Data recording

Table 1 shows the different codes used to denote stillbirth. The delivery service data were recorded in the delivery register with different codes across the facilities. The nurses in-charge of the data recording and collation gave the precise interpretation of the codes used to denote different health indicators in order to avoid misinterpretation errors during data assessment. For instance stillbirth was denoted differently (0, 0/10, 0/0, IUFD, SB, FSB, MSB) across the health facilities but clarified for the purpose of this study by the staff in-charge.

**Table 1:** Different codes used at the facilities.

FACILITY NAME	STILLBIRTH CODES
Facility I	SB; 0; 0/10; Macerated
Facility II	0/10; 0/0
Facility III	SB
Facility IV	SB
Facility V	SB; FSB; IUFD; Macerated SB; MSB
Facility VI	Fresh SB; Stillbirth; MSB; IUFD;
Facility VII	SB
Facility VIII	SB
Facility IX	0; 0/10; Macerated
Facility X	Macerated baby

### Statistical analysis

The quality of the DHIMS-2 database was quantified by assessing the accuracy (error rate) and completeness (percentage of missing data) of the pre-specified neonatal health indicators. A double-visual verification procedure and logic check were applied to estimate the accurate values of the pre-specified health indicators from the primary data. The differences between the estimated values of the health indicators obtained from the primary data and that of the facility and DHIMS-2 data indicated the total number of error in the facility and DHIMS-2 database respectively. Error rates were calculated by dividing the total number of error by the total number of data inspected. Double visual verification is an analogue of double data entry; we applied it because of the inaccuracy of visual verification [6]. The same procedure was also applied to estimate the total number of missing data in the primary data. The percentage of missing data was subsequently calculated by dividing the total number of missing data by the total number of data inspected. The differences between the estimated values of the health indicators in the primary data and that of the DHIMS-2 database were calculated; we subsequently divided the estimated difference (total number of errors) by the total number of data inspected to obtain the error rate and we denoted the estimated error rate as error rate^1^. We repeated this procedure between the primary and facility data; the estimated error rate between both data was denoted as error rate^2^. The same process was applied to estimate the error rate between the facility to DHIMS-2 data and we named the estimated error rate as error rate^3^. All the different error rates estimated are shown in Figure 1.

Completeness was defined as:
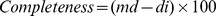



Where ***md*** = total number of missing data; ***di*** = total number of data inspected

The error rate was defined as:
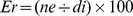



Where ***Er*** = error rate; ***di*** = total number of data inspected

Finally, the 95% confidence interval of the overall missing data (completeness) and error rates were estimated
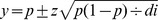



Where ***y*** = 95% confidence interval of the estimate; ***p*** = % of missing data or error rate; ***z*** = 1.645 (1-sided alpha level of 0.5); and ***di*** = total number of data inspected. SPSS (version 20) was used for the analysis [13].

### Ethical approval

This study was conducted in the GAR in conjunction with the Biostatistics Department of the Ghana Health Service which is saddled with the responsibility of collecting, monitoring, managing, and verifying routine GHS clinical data. Written permission to conduct this study in collaboration with the Biostatistics Department of the Ghana Health Service was obtained from the Regional Director of Health Services, Greater Accra Regional Health Directorate, Ghana Health Service. Ethical approval was not required because we only received and analysed anonymous data.

## Results

### Data processing

Thirteen health facilities from seven districts were recruited into the study and a total of 41,000 data recorded on the selected health indicators from January to March 2012. On average 5,800 data entries were inspected per district using logic check and double visual verification procedure. Figure 1 shows the neonatal health indicators considered and the pathway of data flow from the primary data to the DHIMS-2 database. All the health facilities were using a PDC method before uploading their data to the DHIMS-2 database.

After the monthly collation of the primary data from the antenatal, delivery and neonatal admission register by the nurses in their respective departments; data uploading into the DHIMS-2 database was done within the health facilities in all the district hospitals and some of the polyclinics. The facility public health nurses, health information officers and biostatistician were responsible for data uploading depending on the health facility whereas the district public health nurses were uploading data sent from the maternity and health centres.

### Overall completeness of data in the districts and GAR

**Table 2** shows the completeness of the DHIMS-2 data in each district and the GAR. We estimated the percentage of the missing data in the primary data source and in almost all the districts, the percentages of the missing data that were less than 2% with an exception of district D where the value exceeded 25%. Overall percentage of missing data in the GAR was 3.10% (95% C. I = 2.96–3.24).

**Table 2:** Completeness and Accuracy of DHIMS-2 data.

District/Sub-Metro	Completeness (% of missing data)	Accuracy (Error rates in %)
A	1.97	0.22
B	0.09	0.15
C	1.76	3.25
D	25.6	1.27
E	1.27	2.03
F	0	2.66
G	0.88	2.01
**Total Estimate of the DHIMS-2 Data Validity in the GAR**	**3.10% (95% C. I = 2.96–3.24)**	**0.68% (95% C. I = 0.61–0.75)**

### Overall data accuracy in the districts and GAR

Table 2 shows the overall accuracy of the DHIMS-2 data in each district and the GAR. District B had the lowest error rate of 0.15% while most of the districts had an error rate less than 2.1% with the exception of district F and C. The overall error rate of the DHIMS-2 database in the GAR was 0.68% (95% C. I = 0.61–0.75).

### Accuracy of health indicators at the regional level

Estimated error rates of the DHIMS-2 data in the Greater Accra Region (data flow from the primary data source to the DHIMS-2 database) are shown in **Table 3**. The results showed that approximately all the examined health indicators had error rates below 1% except for two parameters: total antenatal registrants and number of babies delivered for which error rates of 1.05% were estimated.

**Table 3:** Error rates of each health indicator in the DHIMS-2 data at the regional level.

GREATER ACCRA REGION (INVOLVING ONLY THE SAMPLED DISTRICTS/SUB-METRO)							
HEALTH INDICATORS	1° DATA	FACILITY DATA	DHIMS – 2 DATA	TOTAL DATA INSPECTED	ERROR RATE^1^ (%)	ERROR RATE^2^ (%)	ERROR RATE^3^ (%)
Total registrants	8984	9079	9079	9012	1.05	1.05	0
Mothers delivered	5903	5875	5912	5925	0.47	0.15	0.62
Babies delivered	5988	5955	5998	6010	0.55	0.17	0.72
Total live birth	5847	5868	5910	6010	0.35	1.05	0.70
Stillbirths	141	87	88	6010	0.90	0.88	0.02
LBW	436	399	399	6010	0.62	0.62	0
Neonatal death	53	52	52	433	0.23	0.23	0
**The overall accuracy of the DHMIS-2 database = 0.68% (95% C. I = 0.61–0.75)**							

**Error rate^1^** represents the percentage of error in the facility data compared to the 1° data.

**Error rate^2^** represents the percentage of error in the DHIMS-2 data compared to the 1° data.

**Error rate^3^** represents the percentage of error in the DHIMS-2 data compared to the facility data.

### Accuracy of health indicators at the district level

**Table 4** shows the estimated error rates of all the health indicators in each of the district as the data flow from the primary data to the DHIMS-2 database. Generally in all the districts, the facility and DHIMS-2 data were almost identical when compared with the exception of district A where facility data differed substantially from the primary and DHIMS-2 data and some of the error rates (error rate^1^ & error rate^3^) exceeded 4%. However, in district A, the primary data were observed to be very similar to the DHIMS-2 data and none of the error rates (error rate^2^) of the health indicators exceeded 0.5%.

**Table 4:** Error rates of each health indicator in the DHIMS-2 data at the district level.

HEALTH INDICATORS	1° DATA	FACILITY DATA	DHIMS – 2 DATA	TOTAL DATA INSPECTED	ERROR RATE^1^ (%)	ERROR RATE^2^ (%)	ERROR RATE^3^ (%)
**DISTRICT A**							
Total registrants	264	265	265	266	0.38	0.38	0
Total delivery	935	897	934	937	4.06	0.11	3.95
Total births	938	894	937	940	4.68	0.11	4.57
Total live birth	933	885	929	940	5.11	0.43	4.68
Stillbirths	7	8	8	940	0.11	0.11	0
LBW	33	36	36	940	0.32	0.32	0
Neonatal death	***	***	***	***	***	***	***
**DISTRICT B**							
Total registrants	1382	1381	1381	1384	0	0	0
Total delivery	543	543	543	545	0	0	0
Total births	545	545	545	547	0	0	0
Total live birth	541	541	541	547	0	0	0
Stillbirths	4	4	4	547	0	0	0
LBW	20	15	15	547	0.91	0.91	0
Neonatal death	7	7	***	1232	0	***	***
**DISTRICT C**							
Total registrants	1474	1455	1455	1478	1.29	1.29	0
Total delivery	975	917	917	979	5.92	5.92	0
Total births	982	926	926	986	5.68	5.68	0
Total live birth	968	915	915	986	5.38	5.38	0
Stillbirths	14	11	11	986	0.30	0.30	0
LBW	48	29	29	986	1.93	1.93	0
Neonatal death	***	***	***	***	***	***	***
**DISTRICT D**							
Total registrants	1448	1453	1453	1452	0.34	0.34	0
Total delivery	536	548	548	540	2.22	2.22	0
Total births	540	552	552	544	2.21	2.21	0
Total live birth	536	551	551	544	2.76	2.76	0
Stillbirths	4	1	1	544	0.55	0.55	0
LBW	65	59	59	544	1.10	1.10	0
Neonatal death	***	***	***	***	***	***	***
**DISTRICT E**							
Total registrants	1362	1421	1421	1364	4.33	4.33	0
Mothers delivered	971	976	976	973	0.51	0.51	0
Babies delivered	986	992	992	988	0.61	0.61	0
Total live birth	957	979	979	988	2.23	2.23	0
Stillbirths	29	13	14	988	1.62	1.52	0.1
LBW	75	49	49	988	2.36	2.36	0
Neonatal death	10	12	***	65	3.08	***	***
**DISTIRCT F**							
Total registrants	766	762	762	770	0.52	0.52	0
Mothers delivered	231	247	247	235	6.81	6.81	0
Babies delivered	233	249	249	237	6.75	6.75	0
Total live birth	232	248	248	237	6.75	6.75	0
Stillbirths	1	1	1	237	0	0	0
LBW	12	12	12	237	0	0	0
Neonatal death	***	***	***	***	***	***	***
**DISTIRCT G**							
Total registrants	2294	2342	2342	2298	2.36	2.36	0
Mothers delivered	1712	1747	1747	1716	2.04	2.04	0
Babies delivered	1764	1797	1797	1768	1.87	1.87	0
Total live birth	1682	1748	1748	1768	3.73	3.73	0
Stillbirths	82	49	49	1768	1.87	1.87	0
LBW	183	199	199	1768	0.90	0.90	0
Neonatal death	53	52	52	433	0.23	0.23	0

**Error rate^1^** represents the percentage of error in the facility data compared to the 1° data.

**Error rate^2^** represents the percentage of error in the DHIMS-2 data compared to the 1° data.

**Error rate^3^** represents the percentage of error in the DHIMS-2 data compared to the facility data.

***denotes data that were not available when the study was conducted.

Further, in other districts the facility and DHIMS-2 data were error-free (error rate^3^) or almost error-free when compared. However, both the facility and DHIMS-2 data were observed to have some degree of discrepancies when compared to the primary data; in district C and F, half of their health indicators had error rates up to 5% and 6% (error rate^1^ and error rate^2^) respectively. In district D, E, and G almost all the error rates (error rate^1^ and error rate^2^) were below 3% whereas in district B almost all the health indicators were error-free.

## Discussion

### Main findings

This study quantified the quality of the DHIMS-2 data by estimating its completeness and accuracy as the data flow from the primary data to the national database. The overall error rate in the DHIMS-2 database was 0.68% (95% C. I = 0.61–0.75) and the percentage of missing data was 3.10% (95% C. I = 2.96–3.24) indicating that the overall accuracy of the DHIMS-2 database was close to an acceptable value of the error rate (0.5%) for high quality data [14], [15]. The accuracy of the DHIMS-2 database was well above the reported average error rates (9.76%) of forty-two source-database validation studies [8] and was observed to be more accurate and complete than a similar database (HMIS database) assessed in Tanzania [9].

It is important to note that there is no consensus on what should be regarded as an acceptable error rate for high quality data; so this value varies across clinical and pharmaceutical fields [6]. The variation in the cut-off point for the acceptable error rate depends on the outcome and the consequences of committing errors. Generally, the majority of the experts agreed that 0.5%, 0–0.1% and 0.2–1%, should be considered as the acceptable error rate for the overall, critical and non-critical variables respectively [14], [15]. Judging from this perspective the overall error rate of the DHIMS-2 data was very close to the acceptable value. However, it is important to emphasise that the final error rate of this data greatly depends on the size of the data inspected. In other words, as the inspected dataset increases the magnitude of the error rate declines.

Overall percentage of missing data in the DHIMS-2 database in each of the districts was negligible with the exception of district D where the percentage of missing data exceeded 25%. This was because one of the facilities in this district was not recording the status of the newborn adequately post-delivery. In all the districts, the facility and DHIMS-2 data were identical or almost identical when compared except for district A where both data differed substantially. The most likely reason for the discrepancy was that the authentic copy of the facility data that was uploaded to the DHIMS-2 database might have been misplaced.

In district B all the three data (primary, facility and DHIMS-2) were almost identical, indicating the ability of the public facilities to provide high quality data. The commonest source of error was inaccurate collation of the primary data; others were inaccurate numbering of the registers, collation of the facility data before the end of the month and inadequate supply of delivery and antenatal register. Other challenges were inadequate training of data collectors (midwives, public health nurses, health information officer and biostatistician), incomplete data capturing, lack of periodic data verification, and more. Variation in coding of health indicators is another important issue that needs attention.

This study evaluated the validity of the DHIMS-2 database and identified plausible sources of errors that should be addressed to improve the quality of the data. At the time of the study, Standard Operating Procedures (SOPs) for the DHIMS-2 database were under development; its application during data acquisition will contribute significantly to the collection of high-quality data. Although introduction of electronic data collection could improve the quality of the database even further the cost associated with electronic data collection may make EDC not a suitable option for low resource settings. Therefore, the focus should be on optimizing PDC procedures, e.g. to implement appropriate quality improvement measures to ensure high quality data. This will require adherence to the SOPs by the data collectors and avoidance of the common sources of errors mentioned earlier.

This study clearly showed that most of the errors in the data were committed during collation of the primary data; indicating that the introduction of double check procedures will reduce the occurrence of errors in the database to a negligible level. This procedure is an analogue of double data entry thus, it is expected to reduce the error rate to 0.001% [16]. Provision of well-designed registers tailored to capture only the required data will enhance uniformity in data capturing processes and accelerate the attainment of high-quality data. Provision of periodic training on data collection will increase staff knowledge and resolve the lack of uniformity in data coding. A concerted effort should be made to integrate more private hospitals and traditional birth attendants into the DHIMS-2 database.

### Study limitations and strengths

The districts involved in this study were randomly sampled and the health facilities that were recruited within the sampled districts were selected based on pre-specified criteria to avoid selection bias. This study recruited about 50% of the districts in the Greater Accra Region in order to have a clearer insight about the quality of the DHIMS-2 data.

It has been reported that the visual verification of data has an inherent weakness of committing 15% error [17]. Thus, we adopted double visual verification; an analogue of double data entry which has been shown to be very sensitive with an error rate of 0.001% [16]. Two people verified the data separately and compared their results in order to resolve any disparity which implies that the probability of committing any error during the verification of the data is directly proportional to the chance that these two assessors will commit the same error. Further, we performed a source – database validation which is in accordance with GCP standard. However, this study only covered the neonatal component of the database; thus it might be argued that the results cannot be generalised to the other components of the database. However, this will only hold grounds if the underlying mechanisms of committing errors in other components of the database are different.

## Conclusion

This study demonstrated that the DHIMS-2 data have a negligible level of missing data while its accuracy was very close to an acceptable standard. It is very clear that the DHIMS-2 data in the GAR can be transformed to high-quality data as demonstrated in district B if other districts can replicate this excellent achievement.
